# Optimal Cut-Off Points on the Health Anxiety Inventory, Illness Attitude Scales and Whiteley Index to Identify Severe Health Anxiety

**DOI:** 10.1371/journal.pone.0123412

**Published:** 2015-04-07

**Authors:** Erik Hedman, Mats Lekander, Brjánn Ljótsson, Nils Lindefors, Christian Rück, Gerhard Andersson, Erik Andersson

**Affiliations:** 1 Karolinska Institutet, Department of Clinical Neuroscience, Division of Psychology, Stockholm, Sweden; 2 Karolinska Institutet, Department of Clinical Neuroscience, Osher Center for Integrative Medicine, Stockholm, Sweden; 3 Karolinska Institutet, Department of Clinical Neuroscience, Division of Psychiatry, Stockholm, Sweden; 4 Linköping University, Department of Behavioural Sciences and Learning, Linköping, Sweden; 5 Stockholm University, Stress Research Institute, Stockholm, Sweden; Univ of Toledo, UNITED STATES

## Abstract

**Background:**

Health anxiety can be viewed as a dimensional phenomenon where severe health anxiety in form of DSM-IV hypochondriasis represents a cut-off where the health anxiety becomes clinically significant. Three of the most reliable and used self-report measures of health anxiety are the Health Anxiety Inventory (HAI), the Illness Attitude Scales (IAS) and the Whiteley Index (WI). Identifying the optimal cut-offs for classification of presence of a diagnosis of severe health anxiety on these measures has several advantages in clinical and research settings. The aim of this study was therefore to investigate the HAI, IAS and WI as proximal diagnostic instruments for severe health anxiety defined as DSM-IV hypochondriasis.

**Methods:**

We investigated sensitivity, specificity and predictive value on the HAI, IAS and WI using a total of 347 adult participants of whom 158 had a diagnosis of severe health anxiety, 97 had obsessive-compulsive disorder and 92 were healthy non-clinical controls. Diagnostic assessments were conducted using the Anxiety Disorder Interview Schedule.

**Results:**

Optimal cut-offs for identifying a diagnosis of severe health anxiety was 67 on the HAI, 47 on the IAS, and 5 on the WI. Sensitivity and specificity were high, ranging from 92.6 to 99.4%. Positive and negative predictive values ranged from 91.6 to 99.4% using unadjusted prevalence rates.

**Conclusions:**

The HAI, IAS and WI have very good properties as diagnostic indicators of severe health anxiety and can be used as cost-efficient proximal estimates of the diagnosis.

## Introduction

Severe health anxiety, throughout this paper defined as DSM-IV hypochondriasis [1], is characterized by a persistent and debilitating fear of somatic illness. For the affected individual the disorder is associated with substantial suffering and for many the problem is chronic [2, 3]. From a societal perspective, identifying persons with severe health anxiety and offering them treatment is important, not the least due to the high costs and strain on health care resources that are associated with the disorder [4, 5]. Although severe health anxiety in form of hypochondriasis is a dichotomous state, i.e. either one has it or not, health anxiety can be viewed as a dimensional phenomenon on which severe health anxiety represents a cut-off where symptoms become clinically significant leading to substantial functional impairment. Therefore, dimensional measures of health anxiety could be used as proximal diagnostic instruments to identify presence of severe health anxiety. Using such measures in self-report format to screen for severe health anxiety has many benefits including that they can be used in clinical settings to save time as not all patients need to undergo an entire diagnostic interview, but only those at risk of having the disorder. Other advantages are that such instruments can be used in epidemiological surveys to estimate prevalence rates in the population and in clinical and research settings it can be of high value to estimate presence of severe health anxiety without needing to use a resource demanding diagnostic interview. This however requires that cut-off points that yield acceptable properties regarding sensitivity, specificity and predictive value can be established on reliable measures.

Three of the most widely used and psychometrically validated dimensional self-report instruments of health anxiety are the Health Anxiety Inventory (HAI) [6], the Illness Attitude Scales (IAS) [7] and the Whiteley Index (WI) [8]. The 14-item WI was developed almost 50 years ago and designed to discriminate persons with severe health anxiety from those not having the disorder [8]. In the present study we used the original WI scale with dichotomous response format. The IAS is comprised of 29 items forming nine subscales and was developed to assess psychopathology related to severe health anxiety [7]. Against the background that neither the WI nor the IAS are fully focused on health anxiety, Salkovskis and co-workers developed the 64-item HAI, which was designed to measure a broad range of health anxiety symptoms and to be sensitive to discriminate persons with elevated health anxiety from somatically ill persons without exaggerated health concerns [6]. The latter means that whereas the IAS has items such as “How often have you been treated the last year?” the HAI as a rule has items phrased as “If I notice an unexplained bodily sensation or change I always try to reassure myself about it”. A person diagnosed with somatic illness could thus in the former case have a high test score without it necessarily reflecting health anxiety. The HAI is thus the most recently developed scale of the three and is based on a cognitive-behavioural model of health anxiety whereas the WI and IAS were developed using a descriptive approach, but all three measures have been shown to have high reliability [6, 8, 9]. Even if the HAI was developed to improve the assessment of health anxiety it should be noted that the WI and IAS have been more widely used in clinical and psychometric research [10].

When it comes to use as instruments to classify presence of severe health anxiety one study investigating the WI found that a score of 8 yielded the best cut-off, but with a rather limited sensitivity of 70% [11]. Other studies have been conducted on the WI as diagnostic screening tool, but have employed an abbreviated 7-item version of the instrument, e.g. [12, 13]. As for the IAS, findings from two studies indicated that a cut-off score of 45 or 51 on the IAS yielded the best cut-off for identifying persons with severe health anxiety [11, 13]. It has been suggested that the bodily preoccupations subscale of the IAS could be used to screen for severe health anxiety due to its brevity [14], but we are not aware of any reliability estimates of this subscale when used as a stand-alone instrument, i.e. when not post-hoc analysed as part of the full scale. When it comes to the HAI, we have found no previous study investigating the scale as a proximal diagnostic instrument, which is a major limitation in this context as the HAI could be viewed as the most well designed health anxiety scale from a theoretical perspective. As reported by Alberts and co-workers [15], a few studies have investigated a short version of the HAI as a screening instrument, but only one has used ROC-analyses in doing so and there is limited data supporting the cut-off scores used in the literature on the short version of the HAI.***2002***).

Brief versions of the instruments are practical when used for screening in clinical settings. However, the rationale for establishing cut-off points is not just to screen for a disorder, but as outlined above also to yield a best estimate of its presence or absence. In many circumstances, such as when conducting treatment research, using reliable self-report instruments to estimate whether criteria for severe health anxiety are met can be a highly cost-efficient alternative compared to a diagnostic interview. In these cases, full version of scales are likely to be more useful due to increased precision as, all other things being equal, measurement error is reduced when scale length is increased [16, 17].

In summary, studies investigating optimal cut-offs of the HAI for identifying severe health anxiety are lacking and no prior study has investigated how the HAI compares to the full versions of the IAS and the WI in this regard. More knowledge in this area could be of high clinical and research utility. The aim of this study was therefore to investigate the HAI, IAS and WI as proximal diagnostic instruments for severe health anxiety.

## Methods

### Design and procedures

This study investigated optimal diagnostic cut-offs on the HAI, IAS and WI using 347 participants of whom 158 (315 were screened) had severe health anxiety in form of DSM-IV hypochondriasis, 97 (314 were screened) had obsessive-compulsive disorder but not severe health anxiety (DSM-IV hypochondriasis), and 92 participants were healthy non-clinical controls, i.e. they had no severe health anxiety or obsessive-compulsive disorder (OCD). Among participants with severe health anxiety 33.5% had a co-morbid anxiety disorder. The corresponding proportions were 24.2% in the OCD sample and 4.1% in the healthy controls. All participants completed assessments with the HAI and IAS and underwent a psychiatric diagnostic assessment interview in which presence or absence of DSM-IV severe health anxiety was established. Assessors were psychiatrists, resident psychiatrists, licensed psychologists or psychology programme students (master’s level) in their final semester under supervision of a licensed psychologist. Only participants with severe health anxiety and healthy controls completed the WI. The OCD sample did not complete the WI for trial-specific practical reasons. The health anxiety instruments were administered via the Internet and participants completed the instruments using their own computers or tablets, which has been shown to be a reliable administration format for the HAI, IAS and WI [18]. The study was conducted at the Karolinska Institutet in Stockholm, Sweden, the instruments were administered in Swedish, and the study was approved by the regional ethics review board in Stockholm. Written electronic informed consent was obtained. Participants with severe health anxiety and OCD took part in clinical treatment trials [19] and did not receive any reimbursement for participation. Healthy controls were recruited through newspaper advertisements and received two movie tickets as compensation.

### Participants

Participant characteristics are presented in Table 1. The study sample comprised adult participants who were recruited from all of Sweden. The main inclusion criteria for the two clinical samples, i.e. participants with severe health anxiety and OCD, were that they had to meet diagnostic DSM-IV criteria for the respective disorder and not meet diagnostic criteria for concurrent psychosis, bipolar disorder or be severely depressed defined as having a score above 30 on the Montgomery Åsberg Depression Rating Scale-Self-rated [20] (severe health anxiety sample) or through clinician-assessment (OCD sample). In addition, participants with OCD should not have a diagnosis of severe health anxiety. A detailed description of the procedures of the clinical trial from which the health anxiety sample was recruited has been previously published [19].

**Table 1:** Description of the participants.

	Severe health anxiety sample	OCD sample	Healthy controls
	n = 158	n = 97	n = 92
Women	125	39	63
Men	33	58	29
Age (SD)	41.5 (13.4)	35.0 (12.9)	48.3 (18.0)
HAI (SD)	104.1 (20.2)	41.6 (19.8)	30.9 (13.5)
IAS (SD)	68.0 (12.0)	29.0 (12.6)	21.2 (8.7)
WI (SD)	10.6 (2.2)	-	1.1 (1.2)

Abbreviations: OCD, Obsessive-compulsive disorder; HAI, Health Anxiety Inventory; IAS, Illness Attitude Scales; WI, Whiteley Index

### Dimensional measures of health anxiety

#### Health Anxiety Inventory

The HAI [6] is a 64-item scale that has two sections, one main section comprised of 47 items measuring cognitive, affective and behavioural aspects of health anxiety and a 17-item “negative consequences” section tapping into the respondent’s perception of how awful it would be to be ill. The full 64-item scale was used in the present study. The HAI was designed to be sensitive in a broad range of health anxiety symptoms and to be effective in identifying participants probable to meet diagnostic criteria for severe health anxiety. In the original article of the scale the authors contrasted the HAI to the WI and IAS and concluded that the latter scales have items not directly related to health anxiety. The total scale range is 0–192 (each item is scored 0–3) and the HAI has been found to have high test-retest reliability (*r* = .90) and convergent and discriminant validity [6].

#### Illness Attitude Scales

The IAS is a 29-item scale that has nine subscales: (I) worry about illness, (II) concerns about pain, (III), health habits, (IV) hypochondriacal beliefs, (V) thanatophobia (fear of death), (VI) disease phobia, (VII) bodily preoccupations, (VIII) treatment experience, and (IX) effects of symptoms. Each item is rated on a 0–4 Likert scale and 27 of the 29 items are used in the total score, which ranges from 0 to108. The scale was designed to tap into psychopathology associated with severe health anxiety and has been shown to have high test-retest reliability (*r* = .89) [21, 22]. When it comes to factor structure, empirical data have however not consistently supported the nine factors suggested by the originators of the scale [23]. As pointed out by Sirri and co-workers the scale was developed to be of high clinical value rather than to adhere to stringent psychometric criteria of item homogeneity [22].

#### Whiteley Index

The 14-item WI [8] was one of the first dimensional measures developed to assess health anxiety and its items are based on clinicians’ experiences of illness characteristics of severe health anxiety. The final items of the scale were selected in part due to their ability to discriminate individuals with severe health anxiety from those without. Pilowsky suggested, based on factor analytic results, that the scale is comprised of three factors, which are bodily preoccupation, disease phobia and disease conviction. Subsequent studies have however shown inconsistent results regarding the factor structure of the WI [24]. In the present study, the version of WI was used that has dichotomous scoring of items (1 or 0) yielding a total score range of 0–14. This scale version was used due to its simplicity, but it should be noted that more recent studies suggest that a version with Likert-scale response options may have better psychometric properties [24]. The WI has been shown to have high test-retest reliability (*r* = .81) and convergent validity [8].

### Diagnostic assessment

The Anxiety Disorders Interview Schedule [25] was used to assess severe health anxiety (DSM-IV hypochondriasis). OCD and other psychiatric axis-I disorders were measured with the Mini International Diagnostic Interview [26]. The diagnostic interviews were conducted over the telephone, which has been shown to be a valid method for conducting psychiatric diagnostic interviews [27].

### Data analysis

Statistical analyses were conducted using STATA 11.0 (STATA inc). Overall differences between the three samples on the HAI, IAS and WI were analysed using ANOVA and post-tests with Bonferroni corrected *p*-values. Sensitivity and specificity were investigated using Received Operating Characteristics (ROC) analyses assessing the Area Under the Curve (AUC) applying the DeLong method for standard error calculation. χ-^2^ tests were used to test potential differences of AUCs between health anxiety measures. From the possible cut-offs on each scale we chose the ones with the highest proportion of correctly classified cases. Where two different cut-offs yielded the same correctly classified proportion of cases, the one with higher sensitivity was chosen. We also conducted analysis of positive and negative predictive values for the optimal cut-off points. As predictive value is highly related to disease prevalence we modelled estimates of predictive value for a range of estimated prevalence rates, which were 2%, 4%, 20%, 25% and 50%. The former two corresponded to prevalence estimates from studies conducted in the general population [4, 28], whereas 20% and 25% corresponded to prevalence rates in medical non-psychiatric clinics as indicated by prior evidence [29]. The extreme of 50% was used as it may correspond to clinical settings where health anxiety problems are very common, such as anxiety clinics. Logistic regression was used to test the association of suggested cut-offs of the HAI, IAS and WI and presence of actual severe health anxiety as assessed with the diagnostic interview.

## Results

### Overall test of differences between groups

Table 1 displays means and SDs of the three health anxiety measures. ANOVA analyses revealed a significant omnibus effect of group (severe health anxiety sample, OCD sample healthy controls) on the HAI and IAS (*F*_(2)_ = 585.6–619.9; *p*<.001) and post-test comparisons showed that all three groups significantly differed from each other with severe health anxiety participants having the highest scores and healthy controls the lowest (*p*<.001). There was also a significant effect of group (severe health anxiety sample vs. healthy controls) on the WI (*F*_(1)_ = 1432.9; *p*<.001).

### Sensitivity and specificity

**Fig 1:**
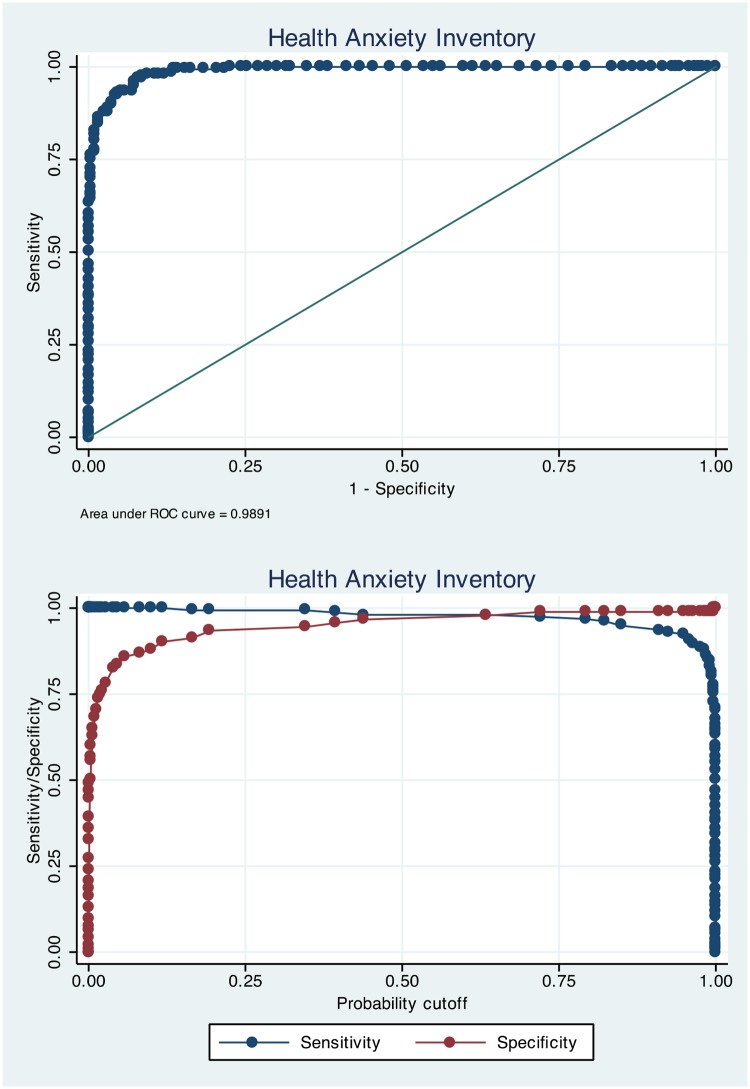
ROC-curve for the Health Anxiety Inventory and sensitivity/specificity as a function of probability cut-off.

**Fig 2:**
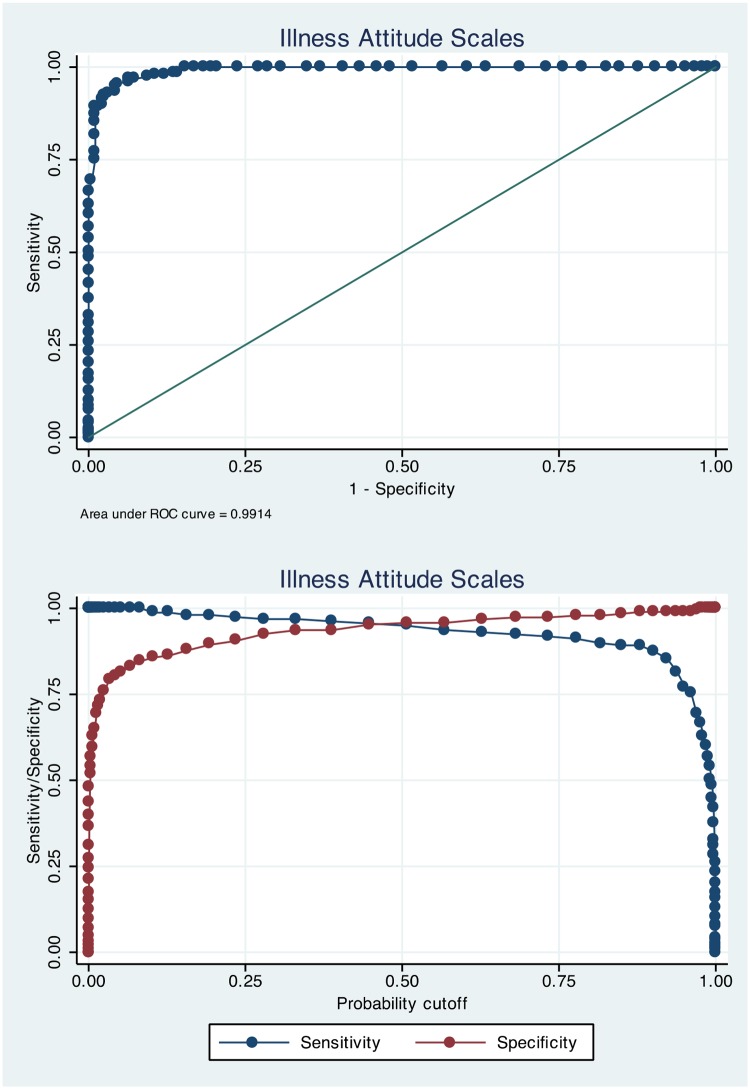
ROC-curve for the Illness Attitude Scales and sensitivity/specificity as a function of probability cut-off.

**Fig 3:**
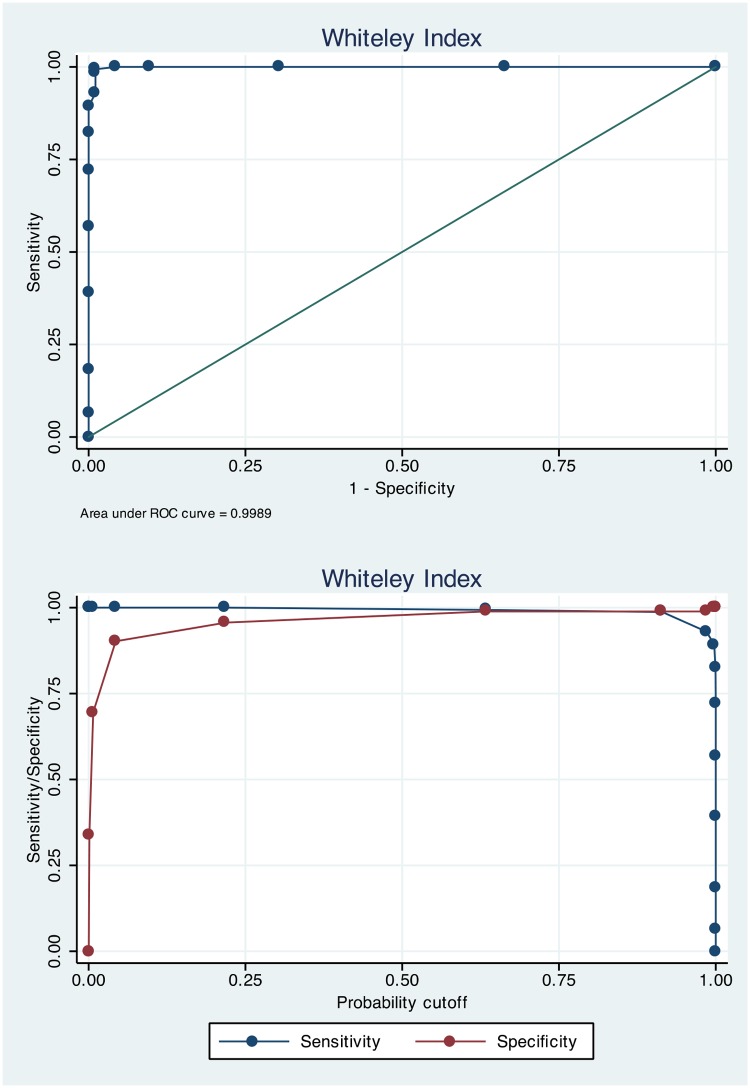
ROC-curve for the Whiteley Index and sensitivity/specificity as a function of probability cut-off.

Table 2 presents sensitivity and specificity estimates for a range of selected scores spanning from 100% sensitivity to 100% specificity on the three health anxiety measures. Figs 1–3 present ROC-curves with AUCs for the HAI, IAS and WI as well as sensitivity and specificity as a function of probability cut-offs. For the HAI, the optimal cut-off was 67 to indicate severe health anxiety, which gave 94.2% correctly classified cases and high sensitivity and specificity. Using this cut-off there was a significant association of test-classification and actual diagnostic status (OR = 316.7; Z = 11.5; *p*<.001). The optimal cut-off score on the IAS was 47, which yielded 95.4% correctly classified cases and high sensitivity and specificity as displayed in Table 2. There was a significant association of test status and actual diagnostic status on the IAS using 47 as cut-off (OR = 431.4; Z = 11.8; *p*<.001). On the WI, the optimal cut-off was 5 which gave 99.2% correctly classified cases and a significant association of classification according to the WI and actual diagnostic status (OR = 14287; Z = 6.74; *p*<.001). It should be noted that the WI analyses were conducted using just the two samples of severe health anxiety participants and healthy controls.

**Table 2:** Sensitivity and specificity for a selection of cuf-off points on the HAI, IAS and WI.

HAI			IAS			WI		
Cut-off	Sensitivity (%)	Specificity (%)	Cut-off	Sensitivity (%)	Specificity (%)	Cut-off	Sensitivity (%)	Specificity (%)
>=			>=			>=		
49	100.00	77.25	38	100.00	84.66	0	100.00	0.00
53	99.37	83.60	40	98.73	86.24	1	100.00	33.70
58	91.10	87.83	42	98.10	89.42	2	100.00	69.57
61	91.10	89.42	44	96.84	93.65	3	100.00	90.22
64	97.47	91.53	46	96.20	93.65	4	100.00	95.65
**67**	**96.20**	**92.59**	**47**	**95.57**	**95.24**	**5**	**99.37**	**98.91**
70	93.67	94.18	48	94.94	95.77	6	98.73	98.91
73	92.41	95.24	50	93.04	96.83	7	93.04	98.91
80	87.97	97.35	54	89.87	97.88	8	89.24	100.00
90	75.95	99.47	60	77.22	98.94	9	82.28	100.00
100	63.29	100.00	63	66.46	100.00	10	72.15	100.00

Abbreviations: HAI, Health Anxiety Inventory; IAS, Illness Attitude Scales; WI, Whiteley Index. Note: estimates for WI based solely on severe health anxiety participants and healthy controls. Note 2: Estimates in bold typeface are the suggested optimal cut-offs.

To test the relative strengths of the HAI and IAS in detecting severe health anxiety we compared their AUCs using ROC-analyses and found no significant difference (χ-^2^_(1)_ = 1.32; *p* = .250).

### Positive and negative predictive value

Table 3 presents positive and negative predictive values for the HAI, IAS and the WI assuming different prevalence estimates of severe health anxiety. As expected, negative predictive values were high for all tested prevalence rates and positive predictive values varied from 21% to 98% where lower prevalence rates produced lower positive predictive values. A comparison of pre-test and post-test odds for having a diagnosis of severe health anxiety based on the lowest tested prevalence rate, 2%, indicated a very high diagnostic utility of the HAI, IAS and WI. The results showed that, for a given individual with an actual diagnosis of severe health anxiety, the odds of having a diagnosis increased from 0.02 using only prevalence data to estimate likelihood of diagnosis to 0.27 (HAI), 0.41 (IAS) and 1.87 (WI) if the individual’s classification scores on the three health anxiety measures were used to predict presence of diagnosis, suggesting an odds increase by 13.5 to 91.0 times.

**Table 3:** Positive and negative predictive values for the suggested cut-offs at different assumed prevalence rates.

HAI Cut-offf >=67			IAS Cut-offf >=47			WI Cut-off >=5		
Prevalence	PPN (%)	NPN (%)	Prevalence	PPV (%)	NPV (%)	Prevalence	PPV (%)	NPV (%)
2%	21.0	99.9	2%	29.1	99.9	2%	65.1	100.0
4%	35.8	99.8	4%	45.5	99.8	4%	79.2	100.0
20%	76.5	99.0	20%	83.4	98.9	20%	95.8	99.8
25%	81.2	98.7	25%	87.0	98.5	25%	96.8	99.8
50%	92.9	96.1	50%	95.3	95.6	50%	98.9	99.4
Study sample	91.6	96.7	Study sample	94.4	96.3	Study sample	99.4	98.9

Abbreviations: HAI, Health Anxiety Inventory; IAS, Illness Attitude Scales; WI, Whiteley Index. Note: estimates for WI based solely on severe health anxiety participants and healthy controls.

## Discussion

The aim of this study was to investigate the HAI, IAS and the WI as proximal diagnostic instruments for severe health anxiety. The results showed that a cut-off of 67 on the HAI, 47 on the IAS and 5 on the WI yielded the best results with sensitivity between 95 and 99% and specificity between 92 and 98%. ROC-analyses revealed that the AUCs were above 98% on all measures and positive and negative predictive values ranged between 21 to 98% depending on modelled prevalence rate. The HAI, IAS and WI thus proved to be very good measures to identify cases of severe health anxiety.

To our knowledge this study is the first to compare the full versions of these three measures in discriminating persons with from those without severe health anxiety. Our analyses showed that they were very similar as proximal diagnostic tools. It is however important to underscore that the slight tendency of the WI to yield higher sensitivity and specificity is partly related to the fact that it was solely based on persons with severe health anxiety and healthy controls. Therefore the data of the present study should not be interpreted as the WI being superior to the HAI and the IAS in this regard.

In comparison to previously conducted studies, Hiller and co-workers [11] found that a cut-off of 45 on the IAS yielded a sensitivity of 72% and a specificity of 79%, thus slightly lower estimates than in the present study. Weck and co-workers [13] found that a cut-off of 50.9 on the IAS yielded a sensitivity of 95% and a specificity of 90%, similar to the present study. As for the WI, a previous study demonstrated that a cut-off of 8 yielded a sensitivity of 70% and specificity of 80% [11]. That cut-off is substantially higher than the suggested 5 of the present study, which gave somewhat more favourable ROC-characteristics. As outlined above this is probably related to the fact that the WI analyses of the present study were conducted on groups at far ends of the health anxiety continuum and the relatively low suggested cut-off is strongly related to the fact that participants without severe health anxiety had very low scores on the WI. In light of this, we suggest that a cut-off of 5 be solely used in the context where it is likely that individuals without severe health anxiety have low levels of psychiatric symptoms. The scales were administered to samples from a Swedish population in Swedish. Scale scores of the persons with severe health anxiety were similar to previously published studies on clinical samples in the UK and US (e.g. [6, 7]), suggesting that study findings are generalizable to a broad cultural context.

As the three measures performed equally it could be argued that WI is preferred over the IAS, which in turn is preferred over the HAI due to differences in scale length. If used solely for screening purposes in clinical settings this is a reasonable view, but in many contexts, such as in health anxiety research, the full HAI and IAS are used due to their good psychometric properties. In such settings, knowledge on optimal cut-offs on these dimensional scales to estimate probability of presence of severe health anxiety is highly useful. So, considering the superior internal consistency of the full HAI and IAS compared to the WI they are likely to be preferred if the aim is to administer a gold-standard self-report instrument that has good diagnostic properties.

Strengths of the present study were the relatively large samples and that a clinical patient group with another principal diagnosis than severe health anxiety, i.e. the OCD sample, was used. From a practical perspective, the Internet-administration of the measures is also a strength as it is likely that future use of these instruments to an increasing extent will be via the Internet. As for limitations, the most important, already mentioned, is that WI analyses were conducted solely on severe health anxiety participants and healthy controls. Another limitation was that we used clinical samples seeking treatment, meaning that it cannot be ruled out that there are differences in the samples compared to the total populations. More research using large samples randomly selected from the populations is therefore needed. A venue for future research is also to investigate diagnostic cut-offs of the short version of the HAI and the WI with Likert-scale response options, and cut-off points for identifying the DSM-5 diagnoses of somatic symptom disorder and illness anxiety disorder.

In spite of these limitations we conclude that the full versions of the HAI, IAS, and WI have very good properties as diagnostic indicators of severe health anxiety including high sensitivity, specificity and predictive value.
